# Are chief executive officers more likely to be first-borns?

**DOI:** 10.1371/journal.pone.0234987

**Published:** 2020-06-30

**Authors:** Cláudia Custódio, Stephan Siegel

**Affiliations:** 1 Finance Department, Imperial College Business School, Imperial College, London, United Kingdom; 2 Department of Finance and Business Economics, Foster School of Business, University of Washington, Seattle, Washington, United States of America; Groupe ESC Dijon Bourgogne, FRANCE

## Abstract

We investigate the link between birth order and the career outcome of becoming Chief Executive Officer (CEO) of a company. CEOs are more likely to be the first-born, i.e., oldest, child of their family relative to what one would expect if birth order did not matter for career outcomes. Both male and female CEOs are more likely to be first-born. However, the first-born advantage seems to largely reflect the absence of an older brother, but not of an older sister. These results are more pronounced for family firms, where traditionally the oldest child is appointed to run the family business, but also hold for non-family firms.

## Introduction

Drucker observes that the common characteristic of CEOs is that they “get the right things done” [1]. While research on CEOs has documented substantial heterogeneity in CEO characteristics, those that make it to the top indeed seem to share superior general ability and better execution skills [2], while being more optimistic and less risk averse than the general population [3]. These characteristics and ultimately an individual’s career outcome are plausibly shaped by a number of innate and pre-determined factors, many of which are still unknown. In this study, we explore an important aspect of CEOs’ rearing environments: birth order and the family composition during childhood.

Birth order has long played a central role for the leadership succession in family firms, reflecting traditional inheritance rules in many countries and cultures that favor the first-born child or, often specifically, the first-born son (male-line primogeniture). However, it remains unclear whether the role of birth-order is specific to family firms or if it can also determine CEO selection in other companies. We conjecture this might be the case. Several studies have documented a negative association between birth-order and IQ as well as educational attainments (see, for example, Behrman and Taubman [4], Black et al. [5], Booth and Kee [6], de Haan [7]). While some between-family studies using U.S. data have failed to find a significant relationship between birth order and IQ (e.g., Damian and Roberts [8]), within-family studies using data from Northern Europe as well as the U.S. have consistently found a significantly negative association (Bjerkedal et al. [9], Black et al. [10], Rohrer et al. [11]). Given these associations between birth order and ability, through IQ and educational attainment, and given that Kaplan et al. [2] identify ability as the primary shared characteristic among those selected for the CEO position, we expect that birth order affects CEO selection also in non-family firms.

Birth order might also affect CEO selection through its possible effect on personality traits and risk preferences, as first proposed by Adler [12]. More recently, Sulloway [13], and Black et al. [14] suggest that first-borns are more likely than later-borns to assume leadership positions as they are more conscientious, ambitious, persistent, and assertive. These traits are consistent with execution skills, the second most important shared characteristic of CEOs identified by Kaplan et al. [2]. However, based on Sullivan’s “Family Niche Theory” later-borns are more adventurous, open to experience, and risk seeking, as they have to find a family niche not yet occupied by older siblings. First-borns might therefore be disadvantaged in becoming CEOs in uncertain or risky environments, or just for the fact that the role implies risk-taking decisions. However, empirical support for the association between birth order and personality traits as well as risk preferences is mixed. While Paulhus et al. [15] and Gilliam and Chatterjee [16] find support for the association of birth order with personality traits and risk preferences respectively, Eckel and Grossman [17], Rohrer et al. [11], Lehmann et al. [18], and Tricarichi and Jalajas [19] do not find any significant associations.

Population based studies, e.g., Kristensen and Bjerkedal [20] and Black et al. [14], suggest that birth-order effects depend on how children “were raised, not how they were born” [21]. Birth-order effects therefore arguably reflect social norms as expressed through different parental attention and investment, and different roles played by older and younger siblings in the same family. Furthermore, to the extent that relevant social norms differ by gender, birth-order effects might also depend on an individual’s gender and that of his or her siblings. Birth-order, as well as family composition, might therefore be an important mechanism through which differences early in life have long reaching consequences.

Since data on top executives’ birth order is not readily available, we collect detailed data on CEOs of small and medium size, privately held U.S. firms, as well as their family structures when growing up. The CEOs in our sample are members of Vistage, a U.S. based executive coaching company that organizes peer advisory groups for executives and business owners. Our main sample consists of about 150 CEOs, who responded to a survey invitation included in Vistage’s monthly newsletter between May and November 2013.

Because our sample exclusively consists of CEOs, we compare the observed frequency of CEOs’ birth order, in other words, the observed proportion of first-borns, second-borns, etc. in our sample of CEOs, with the expected birth-order frequency under the assumption that birth order does not affect the chance of becoming CEO. To avoid confounding effects due to family size, we estimate the expected proportion of each birth order using the number of the CEO’s siblings. We exclude CEOs without siblings, as these CEOs are first- and last-borns at the same time. Given that birth-order effects seem to operate through nurture, not nature (see, e.g., Kristensen and Bjerkedal [20] and Black et al. [14]), we use the total number of siblings, including half-siblings and biologically unrelated siblings, which we obtained from our detailed CEO survey, when determining a CEO’s birth order. We use the term “family birth order” to differentiate our main approach from the “biological birth order,” which reflects the sequence of children of the same biological mother. Similarly, first-born typically refers to the oldest child in the family. While our empirical approach captures the difference between the observed proportion of first-borns and the proportion that is expected if birth order was not associated with the career outcome of CEO, we cannot, with our approach, prove that birth order causes career outcomes. To address this limitation somewhat, we also examine subsets of our data, for which we hypothesize a stronger or weaker birth order effect. Nevertheless, our results remain correlational, not causal in nature.

Using our data about U.S. CEOs and our empirical approach, we address the following three main questions:

- Are CEOs more likely to be first-born than what we would expect if there was no correlation between the career outcome of CEO and birth order?
- Is the age and gender composition of CEOs’ siblings different from what we would expect if there was no correlation between the career outcome of CEO and sibling composition?
- Are CEOs more likely to be first-born than what we would expect if there was no correlation between the career outcome of CEO and birth order in family firms as well as non-family firms?

Our results can be summarized as follows. First, we find that CEOs are significantly more often first-borns than we would expect if there were no association between birth order and being selected as CEO. The average difference between the observed proportion (45%) and the expected proportion (30%) of first-borns in our baseline sample is 15 percentage points (pp).

Second, when we explore differences in gender, we find that both female and male CEOs are more likely to be the oldest child. However, the advantage of being first-born reflects the absence of an older brother but not necessarily the absence of an older sister.

Third, the difference between observed and expected proportion of first-borns is particularly pronounced for CEOs of family firms (27 pp). Consistent with the primogeniture rule being applied but losing importance over time, 79% of older CEOs of family firms are first-borns, while only 44% of younger CEOs of family firms are first-borns. For both groups, the expected proportion of first-borns is 33%. Among non-family firms, we also find that CEOs are more often first-borns than expected in (12 pp). This result is mostly driven by non-founder, outside CEOs, given that for founders this difference is smaller (9 pp) and not statistically significant. This is consistent with risk and uncertainty being more pronounced for start-ups, and the idea that birth order and risk aversion are negatively related.

We perform out-of-sample tests to evaluate the extent to which our finding of a first-born advantage to become CEO is due to sample selection, i.e., the possibility that first-born CEOs are more likely to participate in our survey. First, we use a larger sample of about 600 CEOs, almost all (98%) of which answered an ad-hoc birth-order-related question that was added to a regular survey about the state of the economy. The advantage of this larger data set is that participants did not expect any questions about birth order. However, the disadvantage is that we do not have detailed information about CEOs’ siblings as well as the nature of CEOs’ firms. Second, we use data from Black et al. [14] that are obtained from administrative records. In both cases, sample selection should not affect the results. We find that the overall first-born advantage remains significantly positive, but becomes smaller, varying between 7 and 13 pp, which suggests that our previous results are not simply due to sample selection.

Our study is related to a growing literature examining traits and characteristics of CEOs. Since Bertrand and Schoar [22] initially documented a link between CEO heterogeneity and variation in corporate policies, several studies examine the effects of CEOs’ personal or professional experiences or of specific traits on corporate decisions (see, for example, Faccio et al. [23], Cain and McKeon [24], Custódio and Metzger [25], and Dittmar and Duchin [26]). However, few studies in economics consider becoming CEO as a career outcome per se. Kaplan et al. [2], Graham et al. [3], and Kaplan and Sorensen [27] use data from surveys and executive search assessments of samples of 300 to 3,000 U.S. executives. They find that at the time of selection as CEO, CEOs have superior general skills, lower risk aversion, and higher optimism. Palaiou and Furnham [28] survey about 150 CEOs as well as senior managers of UK firms and document that CEOs are relatively more extraverted, more agreeable, more conscientiousness, and less neurotic. Green et al. [29] perform text analysis of earnings conference calls involving 4,000 U.S. CEOs and CFOs and determine that extraverted CFOs and CEOs are paid more and that extraverted CFOs are more likely to become CEOs. Adams et al. [30] perform predictive regressions of who will become a CEO using assessments from military exams of all Swedish men born between 1951 and 1978. They document significant yet limited predictive power of cognitive and non-cognitive skills as well as height at age 18. Our results suggest birth order, a specific aspect of a CEO’s rearing environment, as a possible determinant of becoming a CEO. Differently from other studies that examine the impact of CEOs’ early life experiences on corporate policies, such as growing up during the Great Depression [31] or experiencing a natural disaster [32], which are common to a cohort of the population, birth order effects reflect variation of the rearing environment within a family and cohort.

Our results are also related to the literature on family firms (see, e.g., Holderness and Sheehan [33], Anderson and Reeb [34], Bertrand et al. [35]). While Smith and Amoako-Adu [36], Villalonga and Amit [37], and Bennedsen et al. [38] examine the succession in family firms, their focus is on the costs and benefits associated with a family member being selected as the new CEO relative to an outsider. We find that the difference between the observed frequency and the expected probability of being first-born is particularly high for family firms relative to non-family firms. Our finding could offer a partial explanation of the negative performance effect associated with family successions, to the extent that it is due to primogeniture or elevated risk aversion of first-borns.

Finally, our study is related to a growing literature on the economic outcomes of birth order and family composition. While earlier research shows that birth border predicts IQ, educational outcome, and earnings (see, e.g., Behrman and Taubman [4], Black et al. [5], and Kristensen and Bjerkedal [20]), our study is part of an emerging literature that specifically studies the effect of birth order on entrepreneurship, leadership, and career outcomes (see, Black et al. [14], Keloharju et al. [39], and Mishkin [40]). Differently from Black et al. [14] and Keloharju et al. [39], who use administrative data from Sweden, we provide evidence from U.S. CEOs. Similar to Black et al. [14], we find that male CEOs in the U.S. are much less likely to have older brothers compared to younger brothers, but equally likely to have older and younger sisters. Importantly, we find a similar effect for female CEOs in the U.S. as well. Understanding the long-run consequences of birth-order is particularly important as birth-order effects are likely the outcome of different roles played by older and younger siblings when growing up as well as of differences in parental attention towards and investment in first- and later-born sons and daughters. Price [41], for example, documents that first-borns receive 20 to 30 more minutes of quality time each day with their parents relative to second-born children. That is, differently from genetic characteristics or general environmental experiences, birth order effects can be shaped by family composition and parenting choices.

The paper proceeds as follows: the next section describes data collection and presents summary statistics; the main results of the paper are then presented in the subsequent sessions; a discussion of the main results and conclusion are presented at the end.

## Data

### Survey of Vistage members

We surveyed CEOs of small and medium size U.S. firms, who were part of the Vistage network, a U.S. based executive coaching company that organizes peer advisory groups for executives and business owners. An invitation to participate in our survey on birth order was included in Vistage’s monthly newsletter between May and November 2013. The newsletter was sent via email to about 12,000 Vistage members, approximately 2,000 of which were CEOs at the time. To participate in the survey, respondents had to click on a member-specific link included in the newsletter that could be used once. The survey was directed at CEOs, and the topic of the survey was disclosed in the newsletter, but all newsletter recipients could participate. After dropping 29 responses from participants that are not CEOs, we are left with 160 survey responses, representing a response rate of 8%, relative to the number of CEOs included among the email recipients, and in line with other recent CEO surveys (see, e.g., Graham et al. [3]). We further drop responses from CEOs of extremely small firms, i.e., firms with less than two employees or less than 1,000 USD of annual revenue (9 observations), as well as responses with incomplete or inconsistent information about birth order and family size (3 observations). Finally, for most of our analysis we exclude CEOs, who grew up without any siblings (7 observations). Our final sample includes responses from 141 CEOs, of which 110 are male and 31 are female. While this small sample serves as our main data set for our empirical analysis below, we also have responses to one survey question about an individual’s birth order from 587 CEOs, who participated in the monthly WSJ/Vistage Small Business CEO Survey in November 2014. Vistage, in collaboration with the Wall Street Journal, conducts a monthly survey among CEOs, who are Vistage members to capture economic conditions from the perspective of small U.S. business. In November 2014, 791 individuals participated in the WJS/Vistage survey, 98% of which responded to the question “What is your birth order?” that Vistage included on our behalf. Based on occupational information, which Vistage provided together with respondents’ year of birth, we identified 587 of the respondents as CEOs. We have no information regarding the overlap between our two samples, as we have no identifiable information on survey participants. The University of Washington Human Subjects Division (HSD) has determined that the research qualifies for exempt status in accordance with the federal regulations under 45 CFR 46.101/ 21 CFR 56.104.

### Summary statistics

Table 1 and S1 Table in the Appendix provide summary statistics for our main data set of 141 CEOs. In our sample, 45.4% of the CEOs are the oldest child in their family. The average number of all siblings, including biological as well as non-biological siblings, is 2.93. For comparison, 53% are first-borns with respect to their biological mother, and the average number of siblings from the same mother is 2.38.

**Table 1:** Summary statistics.

Variable	N	Mean	Std. Dev.	Min	Max
First-born indicator	141	0.45	0.50	0.00	1.00
Number of siblings: All	141	2.93	1.87	1.00	11.00
Male indicator	141	0.78	0.42	0.00	1.00
Current age	138	50.96	9.18	27.00	78.00
Age first became CEO	135	38.64	9.42	19.00	68.00
Founder CEO	139	0.34	0.47	0.00	1.00
Family firm	139	0.22	0.41	0.00	1.00
Outside CEO	139	0.35	0.48	0.00	1.00

This table shows summary statistics for the sample of 141 CEOs. All CEOs in this sample have at least one sibling, including non-biological siblings. *N* denotes the number of observations.

Most CEOs in our sample are male (78%). On average, CEOs are 51 years old in 2012 and first became CEO at the age of 39, suggesting that the carrier outcome of interest (that is, becoming CEO) occurred 12 years before our survey. The average total compensation, including stock options, bonus, and dividends, of the CEOs in the sample is about 430,000 USD in 2012.

In our survey, we ask CEOs about the origin of their relationship with the firm. Based on survey responses we identify 34% of CEOs as founder CEOs, 35% as outside CEOs and 22% as CEOs of family firms. We classify firms as family firms when the CEO indicates that he/she inherited the firm or that the parents, parents-in-law, or family own the firm (excluding firms (co-)founded by the CEO). The average 2012 revenue of the firms run by CEOs in our sample is 24 million USD and the average number of employees is 83. The average ROA as reported by the CEOs for the 2010–2012 period is 9.54%, and the average revenue growth for the same period is 7.12%.

### Are CEOs more likely to be first-borns?

We begin our analysis by comparing the observed birth order distribution of the CEOs in our sample with the distribution of birth order implied by the size of the families in which the CEOs grew up. In other words, our benchmark is the distribution of birth order that we should observe if birth order did not matter for career outcomes. The results in Panel A of Table 2 suggest that 45.4% of CEOs in our sample are first-borns, i.e., the oldest child in the family while growing up, while the expected proportion of being the oldest child, given the size of the family, is only 30.3%. This means that a CEO in our sample is 15 pp more likely to be first-born than we would expect given family size and the assumption that birth order does *not* affect the chances of becoming CEO. The results in Panel A also suggest that CEOs are about 8 pp less likely to be second-borns. A Chi-square test for the difference in distributions between our sample and the distribution implied by family size and CEO assignment, which is independent of birth order, reveals that this difference is statistically significant at the 1% level.

**Table 2:** Baseline results.

**Panel A: Birth order**					
	**Frequency**		**Proportion**		
	**Observed**	**Expected**	**Observed**	**Expected**	**Difference**
Birth order: First	64	42.8	0.454	0.303	0.150
Birth order: Second	32	42.8	0.227	0.303	-0.076
Birth order: Third	24	28.8	0.170	0.204	-0.034
Birth order: Fourth	8	13.8	0.057	0.098	-0.041
Birth order: Fifth	8	6.5	0.057	0.046	0.010
Birth order: Sixth or higher	5	6.3	0.035	0.045	-0.009
Number of observations	141	141			
*Goodness of fit*: *χ*^*2*^	17.08				
*(p-value)*	0.004				
**Panel B: First-born by gender and age**					
	**N**	**Proportion First-born**			***z***
		**Observed**	**Expected**	**Difference**	
All	141	0.454	0.303	0.150	3.885
Male	110	0.473	0.306	0.167	3.805
Female	31	0.387	0.296	0.091	1.112
> 50 years old	64	0.493	0.286	0.207	3.910
< = 50 years old	71	0.415	0.319	0.097	1.670

Panel A shows a chi-square test of the difference in distribution by birth order for the sample of 141 CEOs and the distribution by birth order implied by family size. The sample excludes singletons. Birth order corresponds to the family birth order, i.e., the order of birth of siblings of the same family, including biological siblings, half siblings, and adopted siblings. Panel B shows a test of the observed proportion of first-borns against the expected proportion given the family size, which for a given CEO is calculated as 1/(*n*+1), where *n* is the number of siblings, including non-biological siblings. Panel B reports this test by gender and age of the CEO in 2012. *N* denotes the number of observations, and *z* denotes the *z*-statistic associated with the test that the difference between the observed and expected proportion of first-borns is zero.

In Panel B, we focus on the observed and expected proportion of first-born CEOs, reporting results for all CEOs, by gender and by age. As in Panel A, we calculate the expected proportion of first-borns using the number of all siblings of the CEO. For instance, for a CEO with one sibling the probability of being first-born or the oldest child is 0.50, for a CEO with two siblings it is 0.33, and so on. More formally, for each CEO we calculate the probability of being the oldest child as *P(being the oldest child| number of siblings = n) = 1/(n+1)*, where *n* is the number of siblings of the CEO. We then compare the observed proportion of CEOs, who are first-borns, to the expected proportion, which equals the average probability of being the oldest child. The difference of 15 pp between the observed and expected proportion across all CEOs is statistically significantly different from zero. When repeating the test by gender, we find that the observed proportion of first-born CEOs is larger for man than for woman (47% vs. 39%), as is the difference between observed and expected proportion (17 pp vs. 9 pp). When splitting the sample by CEO age, we find a larger first-born advantage for CEOs above the age of 50 years compared to CEOs, who are 50 years of age or less. This difference could reflect changing social norms that make birth order less important, at least with respect to the career outcome of CEO. An alternative interpretation is that survival rates for CEOs differ by birth-order, with first-borns surviving longer than later-borns.

Since birth order effects were shown to be mostly social as opposed to biological (e.g., Kristensen and Bjerkedal [20]; Black et al. [14]), we focus our baseline results on the total number of siblings, including half-siblings and biologically unrelated siblings. However, when considering biological birth order, that is, only siblings from the same mother, we find results similar to those reported in Table 2 (see S1 Appendix in S2 Table for details). Differently from above, we find a significant first-born advantage for male as well as female CEOs in case of biological birth order. Similarly, to the results in Table 2, we again find a more pronounced first-born advantage for older CEOs.

Overall, we find a significant relationship between birth order and the career outcome of becoming CEO, in particular, that CEOs are more likely to be first-born than they would be if birth order did not affect the likelihood of becoming CEO.

### Family composition

Given the existing evidence that birth order affects cognitive ability through social mechanisms within the family, we explore variation in CEOs’ family structure that might be related to the outcome of being CEO. In particular, we examine family size, as well as the age and gender of siblings. Table 3 shows the results.

**Table 3:** Family composition.

**Panel A: Family size**						
	**N**	**Proportion First-born**				
		**Observed**	**Expected**	**Difference**	***z***	
# of siblings < = 2	73	0.589	0.397	0.192	3.349	
# of siblings >2 and < = 4	47	0.362	0.231	0.131	2.174	
# of siblings >4	21	0.190	0.140	0.050	0.666	
**Panel B: First-female/male-born**						
		**Proportion First-born**				
	**N**	**Observed**	**Expected**	**Difference**	***z***	
First-male-born	110	0.700	0.536	0.164	3.447	
First-female-born	31	0.548	0.578	-0.030	-0.333	
**Panel C: Younger and older brothers and sisters**						
		**Mean**				
		**All**	**Male CEO**	**Female CEO**		
		**(N = 141)**	**(N = 110)**	**(N = 31)**		
Younger brother indicator		0.574	0.573	0.581		
Older brother indicator		0.319	0.300	0.387		
Difference		0.255	0.273	0.194		
(*t*-stat)		*(4*.*041)*	*(3*.*861)*	*(1*.*360)*		
Younger sister indicator		0.461	0.473	0.419		
Older sister indicator		0.404	0.391	0.452		
Difference		0.057	0.082	-0.032		
(*t*-stat)		*(0*.*852)*	*(1*.*084)*	*(0*.*226)*		
Difference in differences		0.199	*0*.*191*	*0*.*226*		
(*t*-stat)		*(2*.*631)*	*(2*.*295)*	*(1*.*270)*		

Panel A shows the observed and expected proportion of first-borns by family size. First-born corresponds to the oldest child among all siblings within a family, including non-biological ones. For a given CEO, the expected proportion is calculated as 1/(*n*+1), where *n* is the number of siblings, including non-biological siblings. The number of siblings includes all siblings within a family, including non-biological ones. Panel B shows the observed and expected proportion of first-female and first-male born CEOs. Panel C reports the observed proportion of CEOs with younger and older siblings by gender. *N* denotes the number of observations, and *z* denotes the *z*-statistic associated with the test that the difference between the observed and expected proportion of first-borns is zero.

Family size can confound birth order effects. For example, if CEOs are more likely to come from smaller families, the proportion of first-borns would be larger among CEOs than in the general population. We use the actual number of a CEO’s siblings to estimate the expected proportion of being first-born, such that family size or correlated family characteristics do not affect our previous results. However, it is possible that the birth order effect varies with family size. For example, in larger families parents might place more importance on the oldest child, however, there might also be fewer resource per child to begin with lowering the absolute effect of preferential of the oldest child.

The evidence in Panel A of Table 3 suggests that being first born is more important in smaller families. The difference between the observed and expected proportion of first-borns is largest for CEOs from families with at most three children (19 pp) and smallest, and insignificant, among CEOs from families with six or more children (5 pp). Being first-born seems to provide an advantage to become CEO as long as family size is not too large.

This result also mitigates concerns that our results are biased because family size increases with the “quality” of the first-born child. Specifically, assume the first child’s abilities exceed the parents’ expectations and hence lead to more children than the parents would have had otherwise. If parents were correct, and the first child exceeds the abilities of the younger siblings, the difference between observed and expected proportion of first-borns statistic would be biased upwards as the expected probability of being first-born would be lower (due to the larger number of siblings) exactly in those cases where the first-born child has superior skills and is more likely to become a CEO. If this mechanism was at work, we should observe a larger first-born advantage as family size increases, the opposite of the results in Panel A of Table 3.

In Panel B, we address another question related to family composition: How does the first-born effect interact with gender? That is, are male (female) CEOs more likely to be the first-male-born (first-female-born) child in their families than what we would expect given the number of male (female) siblings? We find that male CEOs are 16 pp more likely than expected to be the first-male-born child of their families. However, we do not find this to be the case for female CEOs. That is, on average the female CEOs in our main sample are the first-female-born child in their families as often as we would expect, given the number of their female siblings and the assumption that birth order does not affect the chance of becoming CEO.

In Panel C, we further explore differences of birth order effects related to gender. Specifically, we compare the proportion of CEOs that have a younger brother to the proportion of CEOs that have an older brother. Similarly, we compare the proportion of CEOs that have a younger sister to the proportion of CEOs that have an older sister. While the existence of an older brother is 26 pp less likely than the existence of a younger brother, the difference between the existence of an older sister and a younger sister is only 6 pp and statistically insignificant. Importantly, the difference between these two differences (20 pp) is statistically significant. That is, CEOs are significantly more likely to have a younger vs. older brother compared to a younger vs. older sister. Interestingly, we find the same pattern for male and female CEOs, i.e., older brothers are relatively less likely than older sisters, by 19 pp for male CEOs and by 23 pp for female CEOs. However, given the small number of female CEOs, this difference is not statistically significant in the case of female CEOs. Said differently, an older brother, but not an older sister, seems to reduce the chances of both later-born sons and daughters to become CEOs.

Overall, being first-born seems to play a more important role among smaller families. We also find that male CEOs are more likely to be the oldest male, while female CEOs are not more likely to be the oldest female. Finally, we show that CEOs are much more likely to have younger vs. older brothers compared to younger vs. older sisters.

## Family and non-family firms

### Family firms

For family firms, traditional succession norms favor the oldest child, in particular the oldest son (see, for instance, Bennedsen et al. [38]). As expected, we find that CEOs are particularly more likely to be first-born in family firms compared to all other firm types. Table 4 presents the results.

**Table 4:** Family firms.

**Panel A: Family firms**					
		**Proportion First-born**			
	**N**	**Observed**	**Expected**	**Difference**	***z***
All	30	0.600	0.330	0.270	3.145
Male	23	0.609	0.331	0.278	2.828
Female	7	0.571	0.326	0.245	1.384
> 50 years old	13	0.786	0.330	0.456	3.629
< = 50 years old	16	0.438	0.330	0.107	0.913
**Panel B: First female/male born**					
		**Proportion First-born**			
	**N**	**Observed**	**Expected**	**Difference**	***z***
First-male-born	23	0.783	0.516	0.267	2.559
First-female-born	7	0.571	0.619	-0.048	-0.259
**Panel C: Younger and older brothers and sisters**					
		**Mean**			
		**All**	**Male CEO**	**Female CEO**	
		(N = 30)	(N = 23)	(N = 7)	
Younger brother indicator		0.733	0.739	0.714	
Older brother indicator		0.200	0.217	0.143	
Difference		0.533	0.522	0.571	
(*t*-stat)		*4*.*287*	*3*.*761*	*1*.*922*	
Younger sister indicator		0.467	0.478	0.429	
Older sister indicator		0.300	0.261	0.429	
Difference		0.167	0.217	0.000	
(*t*-stat)		*1*.*223*	*1*.*417*	*0*.*000*	
Difference in differences		0.367	*0*.*304*	*0*.*571*	
(*t*-stat)		*2*.*165*	*1*.*499*	*1*.*922*	

Panel A shows the observed and expected proportion of first-borns CEOs in family firms, by gender and by age. First-born corresponds to the oldest child among all siblings within a family, including non-biological ones. For a given CEO, the expected proportion is calculated as 1/(*n*+1), where n is the number of siblings, including non-biological siblings. Panel B shows the observed and expected proportion of first-female and first-male born CEOs in family firms. Panel C reports the observed proportion of CEOs with younger and older siblings by gender, in family firms. The sample excludes singletons. *N* denotes the number of observations, and z denotes the z-statistic associated with the test that the difference between the observed and expected proportion of first-borns is zero.

Panel A of Table 4 shows that the difference between the observed and expected probability of being the oldest child is 27 pp across all family firms, and 46 pp for family firms, when the CEOs is more than 50 years old. The difference is only slightly more pronounced for male (28 pp) than for female CEOs (25 pp).

In Panel B, we repeat the analysis of first-male-born and first-female-born in the context of family firms. We find that 78% of male CEOs in family firms are the first male-born children in their family, against an expected proportion of 52%. This difference of 27 pp is statistically significant. For women, we again do not find a significant difference between the observed and expected proportions of first-female-born CEOs in family firms.

In Panel C, we further explore the family structure of CEOs of family firms. Both male and female CEOs are more likely to have younger brothers when compared to older ones, than they are to have older sisters when compared to younger ones. This difference-in-differences is larger for family firms at 37 pp than the baseline result at 20 pp. This difference is also more pronounced from females in family firms at 57 pp.

In general, our results are more pronounced for family firms, consistent with the traditional social rule of appointing the oldest son to run the family business. In the next section we focus on non-family firms to understand if the effect of birth order exists in firms where this succession rule is not applicable.

### Non-family firms

We repeat our baseline tests in the sub-sample of non-family firms. Table 5 shows these results. The difference between observed and expected proportion of first-borns for CEOs in non-family firm is 12 pp and statistically significant. As before, the difference is more pronounced for male CEOs and for older CEOs. Among CEOs of non-family firms, we are able to identify founder CEOs based on CEOs’ survey responses. Founder CEOs are particularly interesting because two mechanisms associated with birth order, cognitive ability and risk aversion, might be at play. On the one hand, and consistent with the results for the overall sample, founder CEOs can be more likely to be of lower birth order because starting and developing a business requires cognitive ability and talent. This is consistent with Adams et al. [42], who find a positive effect of founder CEOs on firm performance. On the other hand, starting a firm is risky, so that we should expect founder CEOs to be less risk averse and therefore of a higher birth order. We find that founder CEOs are less likely to be first-borns than CEOs of family firms or outside CEOs who were appointed or promoted by the firm. In fact, the difference between observed proportion and expected proportion is only 9 pp and not statistically significant for founders. This is in line with later-born siblings being less risk averse than first-borns and for this reason more likely to start their own company. For outside CEOs, we find that the first-born advantage among these CEOs is of 15 pp and statistically significant.

**Table 5:** Non-family firms.

**Panel A: All nonfamily firms**					
	**N**	**Proportion First-born**			
		**Observed**	**Expected**	**Difference**	***z***
All nonfamily firms	109	0.422	0.299	0.123	2.800
Founder CEO	47	0.383	0.292	0.091	1.372
Outside CEO	48	0.458	0.304	0.154	2.326
**Panel B: First female/male born**					
	**N**	**Proportion First-born**			
		**Observed**	**Expected**	**Difference**	***z***
First-male-born	85	0.694	0.549	0.146	2.697
First-female-born	24	0.542	0.566	-0.024	-0.240
**Panel C: Younger and older brothers and sisters**					
		**Mean**			
		**All**	**Male CEO**	**Female CEO**	
		(N = 109)	(N = 85)	(N = 24)	
Younger brother indicator		0.532	0.529	0.542	
Older brother indicator		0.339	0.306	0.458	
Difference		0.193	0.224	0.083	
(*t*-stat)		*2*.*677*	*2*.*762*	*0*.*526*	
Younger sister indicator		0.468	0.482	0.417	
Older sister indicator		0.422	0.412	0.458	
Difference		0.046	0.071	-0.042	
(*t*-stat)		*0*.*600*	*0*.*815*	*0*.*253*	
Difference in differences		0.147	*0*.*153*	*0*.*125*	
(*t*-stat)		*1*.*721*	*1*.*655*	*0*.*592*	

Panel A shows Panel A shows the observed and expected proportion of first-borns CEOs in non-family firms, by gender and by age. First-born corresponds to the oldest child among all siblings within a family, including non-biological ones. For a given CEO, the expected proportion is calculated as 1/(*n*+1), where n is the number of siblings, including non-biological siblings. Panel B shows the observed and expected proportion of first-female and first-male born CEOs in non-family firms. Panel C reports the observed proportion of CEOs with younger and older siblings by gender, in non-family firms. The sample excludes singletons. *N* denotes the number of observations, and *z* denotes the z-statistic associated with the test that the difference between the observed and expected proportion of first-borns is zero.

In Panels B and C, we repeat the analysis of the family structure. We find that male CEOs in non-family firms are also significantly more likely to be the first-born son than what would be predicted by their family structure, but this is not the case for female CEOs that are not more likely to be the first-born female in their families. In this sample of non-family firms, we also observe the ‘absence of an older brother advantage’ effect, though in this sample this is only present for male CEOs. In other words, male CEOs are less likely to have older brothers than to have younger ones, but this difference is much less pronounced and not significant for older sisters when compared to younger ones.

Overall the tests on non-family firms suggest that birth order plays a role in becoming CEO beyond the traditional rule of appointing the oldest son (child) to run the family business. The results from family and non-family firms combined also suggest that to the extent that entrepreneurship is transmitted intergenerationally (see, e.g., Charles and Hurst [43]), the uniform transmission through family culture or genes is not strong enough to dominate the differential effects of birth order.

## Discussion

### CEOs’ perceptions of the importance of birth-order

Our results so far are consistent with birth-order influencing career paths and the outcome of getting to the top. We asked the CEOs in our sample about their perception of the importance of birth order for their career outcome. Table 6 shows the results. The majority, 56% of the CEOs, believe that birth order has contributed to them becoming CEOs. Interestingly, 71% of first-borns believe that birth order mattered, while only 43% of later-borns believe so. While consistent with our baseline result, it is possible that first-born CEOs rationalize their career success with their birth order. However, given overconfidence and self-attribution bias, such rationalization of a generally successful outcome (being CEO) appears unlikely. Indeed, when using regression analysis to explain these perceptions, we find that male CEOs are significantly less likely to attribute their career outcome to birth order, consistent with overconfidence and self-attribution bias being particularly pronounced among men relative to women. We also find that CEOs who achieved their CEO position later in their careers as well as CEOs with a college degree, perceive birth order as less important. Finally, consistent with our findings above, CEOs of family firms are also more likely to have this perception.

**Table 6:** Perceived importance of birth order.

**Panel A: By birth order**			
	N	Yes	No
CEOs: All	132	56%	44%
CEOs: First-borns	62	71%	29%
CEOs: Later-borns	70	43%	57%
*Independence*: *χ*^*2*^	10.55		
*(p-value)*	*0*.*001*		
**Panel B: Regression**			
	(1)	(2)	
First-born	0.281*** (0.083)	0.305*** (0.092)	
Male		-0.180* (0.101)	
Current age		0.001 (0.005)	
Number of siblings		0.029 (0.077)	
Number of siblings sq.		-0.002 (0.008)	
College degree		-0.183 (0.114)	
Age first became CEO		-0.011** (0.005)	
Family firm indicator		0.076 (0.103)	
Constant	0.429*** (0.060)	1.034*** (0.308)	
Observations	132	122	
Adjusted R-squared	0.073	0.108	

This table shows the results from the survey question: “Do you believe your birth order contributed to your becoming CEO or, if not the CEO, obtaining your current position?” *N* denotes the number of observations.

Panel B reports OLS results from a linear probability model where the dependent variable is an indicator variable equal to one if the CEO answered yes to the question above and zero otherwise. Heteroskedasticity-robust standard errors are reported in parentheses.

The difference between first- and later-borns in their perceptions of the importance of birth order might raise the concern that we might have oversampled first-born CEOs, since we specifically ask them to answer a survey about birth-order, and they seem to be more aware of potential effects of birth order. We address this concern with two out-of-sample tests.

### Out-of-sample evidence

In the first out-of-sample test, we use birth order information for 587 CEOs that participated in the WSJ/Vistage Small Business CEO Survey in November 2014. While this larger sample is virtually free of sample selection concerns given a response rate of 98%, we only have data on CEOs’ biological birth order, i.e., whether they were the first, second, third and so on child of their biological mother and their year of birth, but no additional information.

Importantly, we do not know how many siblings a CEO has. Therefore, we cannot drop CEOs without siblings, nor can we calculate the expected probability of being first-born given the number of siblings. Instead, we use data for the U.S. population as benchmark, corresponding to a CEO’s year of birth. Specifically, we obtain the proportion of live births by (biological) birth order in a given year from the “Vital Statistics Birth Data” of the National Center for Health Statistics (NCHS). Data from 1968 to 2015 is obtained through NBER, data from 1940 to 1967 is obtained from the National Center for Health Statistics reports. We assume that the birth order distribution prior to 1940 is the same as the one in 1940. We then compare the birth order distribution observed in our CEO sample with the birth order distribution observed for the U.S. population. Table 7 Panel A reports the comparison between the two distributions, revealing a significant (*p*-value: 0%) difference between both distributions.

**Table 7:** Out of sample evidence using larger CEO sample.

**Panel A: Birth order**					
	Frequency		Proportion		
	Observed	Expected	Observed	Expected	Difference
Birth order: First	244	186	0.416	0.318	0.098
Birth order: Second	173	156	0.295	0.267	0.028
Birth order: Third	96	103	0.164	0.175	-0.011
Birth order: Fourth	37	59	0.063	0.101	-0.038
Birth order: Fifth	22	33	0.037	0.056	-0.018
Birth order: Sixth or higher	15	50	0.026	0.084	-0.059
Number of observations	587	587			
*Goodness of fit*: *χ*^*2*^	55.89				
*(p-value)*	0.000				
**Panel B: First-born by gender and age**					
		Proportion First-born			
	N	Observed	Expected	Difference	*z*
All	587	0.416	0.318	0.097	5.099
Male	520	0.412	0.318	0.093	4.557
Female	67	0.448	0.312	0.136	2.401
> 50 years old	475	0.385	0.285	0.101	4.856
< = 50 years old	238	0.454	0.370	0.084	2.674

This table shows the baseline test for a sample of 587 CEOs. The sample includes singletons, and first-born corresponds to the oldest child among the siblings of the same biological mother. For a given CEO the expected proportion of first-born is given by the birth order distribution of the U.S. Population based on the year of birth of the CEO. Panel A shows a chi-square test of the difference in the observed and expected distributions by birth order. Panel B shows a test of the observed proportion of first-borns against the expected proportion. *N* denotes the number of observations, and z denotes the *z*-statistic associated with the test that the difference between the observed and expected proportion of first-borns is zero.

Panel B further shows a statistically significant difference between the observed and the expected proportion of first-borns among all CEOs (10 pp) as well as among female (14 pp) and male CEOs (9 pp).

While the out-of-sample first-born advantage is smaller than in our smaller data set, the comparison is not straightforward given the differences between both approaches. For comparability between the large and small sample results, we therefore reproduce corresponding results for our small sample. That is, we use biological birth order, we include CEOs without siblings, and we use U.S. population benchmarks. We report these results in S1 Appendix in S3 Table. The comparable first-born advantage in our small sample is 21 pp.

In our second out-of-sample test, we use summary statistics and regression results reported by Black et al. [14] for data from Swedish population registers to implement the equivalent to our baseline tests in their sample. Their sample includes only males from families with at least two male children. We compare the observed proportion of first-born CEOs (top managers in their designation) to the proportion implied by the population statistics. In S1 Appendix in S4 Table, we show in detail how we make inference about the observed and implied proportion of first-born CEOs using summary data from Black et al. [14]. In Table 8, we report a difference between the observed and expected proportion of first-borns of 11 pp. Using reported regression results, we adjust this difference for the effect of family characteristics, such as family size, which are controlled for by family fixed effects. Doing so, we obtain a more conservative estimate of 8 pp for the difference between the observed and expected proportion of first-borns.

**Table 8:** Out of sample evidence using data from Black et al. [14].

	N	Proportion First-born			*z*
		Observed	Expected	Difference	
First-born Male	3,917	0.479	0.369	0.110	14.267
First-born Male (adjusted for family f. e.)	3,917	0.447	0.369	0.079	10.117

This table shows the baseline test for a sample of 3,917 male CEOs (top managers) from Black et al. [14]. The sample excludes singletons, and a first-born corresponds to the oldest child among the siblings of the same biological mother. For a given CEO, the expected proportion of first-born is given by the birth order distribution in Black et al.’s [14] sample of 727,111 males. *N* denotes the number of observations, and z denotes the *z*-statistic associated with the test that the difference between the observed and expected proportion of first-borns is zero.

Overall, our out-of-sample test results confirm that CEOs are more likely to be the oldest child in their families. However, they also suggest that the effect might be smaller than what it is measured in our original birth-order survey, possibly due to sample selection. Nevertheless, it is unclear that sample selection would affect our results comparing birth order effects across different subsets of CEOs within our sample.

### Birth order and firm performance

Our results suggest that CEOs are more likely first-borns than what we would expect if birth order did not matter for future career outcomes. The result is consistent with the previously documented higher cognitive and non-cognitive skills of first-borns. But does the relative advantage of first-born children becoming CEOs imply a relationship between CEO birth order and firm performance? We do not find any evidence that first-born CEOs perform better, in terms of their firms’ ROA or sales growth, or earn higher incomes compared to later born CEOs. It is possible that our sample is too small to detect performance differences across CEOs. However, these results are also consistent with first-borns having an advantage of being selected as CEOs, while selection ensures comparable skills among all those selected, first-borns as well as later-borns.

## Conclusions

Analyzing a sample of CEOs of small and medium companies in the U.S., we identify birth order as a significant determinant of becoming CEO. The CEOs of firms in our sample are significantly more likely to be first-born, i.e., the oldest child in the family when growing up, than what is expected given the number of their siblings. The first-born effect is particularly pronounced for family firms, which traditionally appoint the oldest child to run the family business, however family firms do not solely explain our results. These results suggest that birth-order effects are present beyond the traditional rule in family firms of appointing the oldest children to manage the family business.

For male and female CEOs, the first-born advantage seems to largely reflect the absence of an older brother, not of an older sister. Consistent with changing family structures and, in particular, rearing practices and social norms, we also document that the first-born effect is more pronounced for older CEOs.

We address concerns about over-sampling first-born CEOs in our main sample by looking at two larger data sets that are essentially free from sample selection. We find a smaller, yet still economically large and statistically significant effect in both larger data sets. Finally, we also note that concerns about sample selection in our main sample are less likely to affect our cross-sectional results, such as differences across firm types, cohorts, or rearing environments.

Overall, our results suggest that individuals’ early life experiences, as captured by the rearing environment and the parenting choices related to their birth order, can have a long-lasting impact on career outcomes, such as becoming the CEO of an organization.

## Supporting information

S1 Appendix Internet appendix. (DOCX)
