# Supplementary material for: Are chief executive officers more likely to be first-borns?
Source: PLoS One. 2020 Jun 30;15(6):e0234987. doi: 10.1371/journal.pone.0234987 (PMC7326194; doi:10.1371/journal.pone.0234987)
Supplement: S1 Appendix — (DOCX) [file pone.0234987.s001.docx]

Are chief executive officers more likely to be first-borns?

Supporting Information

Cláudia Custódio

Imperial College Business School

c.custodio@imperial.ac.uk

Stephan Siegel

University of Washington

ss1110@uw.edu

Internet Appendix

**Table S1. Additional sample description – performance variables.**

| **Variable** | **N** | **Mean** | **Std. Dev.** | **Min** | **Max** |
| --- | --- | --- | --- | --- | --- |
| CEO’s income (2012, in million USD) | 137 | 0.34 | 0.38 | 0.05 | 1.55 |
| Revenue (2012; in million USD) | 140 | 24.18 | 32.99 | 1.50 | 150.00 |
| Number of employees (FTE) | 140 | 82.66 | 101.14 | 2.00 | 450.00 |
| ROA (3-year average, 2010-12, in %) | 117 | 9.54 | 6.51 | 0.00 | 25.00 |
| Revenue growth (3-year CAGR, 2010-12, in %) | 132 | 7.12 | 20.79 | -57.50 | 52.50 |

**Table S2. Biological Birth Order Excluding Singletons.**

| **Panel A: Birth order** |  |  |  |  |  |
| --- | --- | --- | --- | --- | --- |
|  | **Frequency** | | **Proportion** | | |
|  | **Observed** | **Expected** | **Observed** | **Expected** | **Difference** |
| Birth order: First | 65 | 44 | 0.485 | 0.327 | 0.159 |
| Birth order: Second | 31 | 44 | 0.231 | 0.327 | -0.095 |
| Birth order: Third | 21 | 26 | 0.157 | 0.196 | -0.039 |
| Birth order: Fourth | 7 | 11 | 0.052 | 0.081 | -0.029 |
| Birth order: Fifth | 7 | 4 | 0.052 | 0.033 | 0.019 |
| Birth order: Sixth or higher | 3 | 5 | 0.022 | 0.037 | -0.014 |
| Number of observations | 134 | 134 |  |  |  |
| *Goodness of fit:* χ*^2^* | 18.74 |  |  |  |  |
| *(p-value)* | 0.002 |  |  |  |  |
| **Panel B: First-born by gender and age** | | |  |  |  |
|  | **N** | **Proportion First-born** | | | ***z*** |
|  |  | **Observed** | **Expected** | **Difference** |  |
| All | 134 | 0.485 | 0.327 | 0.159 | 3.914 |
| Male | 104 | 0.481 | 0.327 | 0.154 | 3.348 |
| Female | 30 | 0.500 | 0.326 | 0.174 | 2.039 |
| > 50 years old | 70 | 0.514 | 0.303 | 0.212 | 3.852 |
| <= 50 years old | 62 | 0.452 | 0.350 | 0.101 | 1.670 |

Panel A shows a chi-square test of the difference in distribution by birth order for the sample of 134 CEOs and the distribution by birth order implied by the number of biological siblings of the same mother. The sample excludes singletons. Birth order corresponds to the biological birth order, i.e., the order of birth of siblings of the same mother, excluding half siblings, and adopted ones. Panel B shows a test of the observed proportion of first-borns against the expected proportion given the number of siblings of the same mother, which for a given CEO is calculated as 1/(*n*+1), where *n* is the number of biological siblings of the same mother. *N* denotes the number of observations, and *z* denotes the *z*-statistic associated with the test that the difference between the observed and expected proportion of first-borns is zero.

**Table S3. Biological Birth Order Including Singletons.**

| **Panel A: Birth order** |  |  |  |  |  |
| --- | --- | --- | --- | --- | --- |
|  | **Frequency** | | **Proportion** | | |
|  | **Observed** | **Expected** | **Observed** | **Expected** | **Difference** |
| Birth order: First | 77 | 47 | 0.531 | 0.326 | 0.205 |
| Birth order: Second | 30 | 39 | 0.207 | 0.268 | -0.061 |
| Birth order: Third | 21 | 25 | 0.145 | 0.171 | -0.026 |
| Birth order: Fourth | 7 | 14 | 0.048 | 0.098 | -0.050 |
| Birth order: Fifth | 7 | 7 | 0.048 | 0.052 | -0.004 |
| Birthorder: Sixth or higher | 3 | 12 | 0.021 | 0.084 | -0.063 |
| Number of observations | 145 | 145 |  |  |  |
| *Goodness of fit:* χ*^2^* | 31.88 |  |  |  |  |
| *(p-value)* | 0.000 |  |  |  |  |
| **Panel B: First-born by gender and age** | | |  |  |  |
|  |  | **Proportion First-born** | | |  |
|  | **N** | **Observed** | **Expected** | **Difference** | ***z*** |
| All | 145 | 0.531 | 0.326 | 0.205 | (5.270) |
| Male | 114 | 0.535 | 0.326 | 0.209 | (4.762) |
| Female | 31 | 0.516 | 0.325 | 0.191 | (2.267) |
| > 50 years old | 74 | 0.541 | 0.292 | 0.249 |  |
| <= 50 years old | 71 | 0.521 | 0.362 | 0.16 |  |

Panel A shows a chi-square test of the difference in distribution by birth order for the sample of 145 CEOs and the distribution by birth order implied by the US population in the year of birth of the CEO. The sample includes singletons. Birth order corresponds to the biological birth order, i.e., the order of birth of siblings of the same mother, excluding half siblings, and adopted ones. Panel B shows a test of the observed proportion of first-borns against the expected proportion given the birth order distribution in the US population in the year when the CEO was born. *N* denotes the number of observations, and *z* denotes the *z*-statistic associated with the test that the difference between the observed and expected proportion of first-borns is zero.

**Table S4. Inference Using Swedish Males.**

|  | **First  child** | **Second  child** | **Third  child** | **Fourth  child** | **Fifth  child** | **Sum** | **Reference** |
| --- | --- | --- | --- | --- | --- | --- | --- |
| % CEOs | 0.007 | 0.005 | 0.004 | 0.003 | 0.002 |  | Table 2: Cols 2 - 6 |
| Population | 267,923 | 271,373 | 132,665 | 44,108 | 11,042 | 727,111 | Table 2: Cols 2 - 6 |
| CEOs | 1,875 | 1,357 | 531 | 132 | 22 | 3,917 | Calculated |
| Proportion of CEOs that are first-born - Observed | 0.4788 | 0.3464 | 0.1355 | 0.0338 | 0.0056 | 1.0000 |  |
| Proportion of CEOs that are first-born - Expected | 0.3685 | 0.3732 | 0.1825 | 0.0607 | 0.0152 | 1.0000 |  |
| Difference in % CEO relative to first child: | | | | | | | |
| Population numbers |  | -0.0020 | -0.0030 | -0.0040 | -0.0050 |  | % CEO above |
| Regression based, controlling for family fixed effects |  | -0.0017 | -0.0020 | -0.0018 | -0.0026 |  | Table 4: Col 3 |
| Adjusted % CEO | 0.0065 | 0.0048 | 0.0045 | 0.0047 | 0.0039 |  |  |
| CEOs (implied) | 1,751 | 1,312 | 602 | 209 | 43 | 3,917 |  |
| Proportion first-born (implied) | 0.4470 | 0.3350 | 0.1536 | 0.0533 | 0.0111 | 1.0000 |  |

This table shows the inference using data from Swedish Males from Black et al. [14]. The sample includes 727,111 males, of which 3,917 are CEOs (top managers).
